# Lifestyle-Based Interventions for Major Depressive Disorder and Their Role Alongside Antidepressant Treatment: A Systematic Review

**DOI:** 10.7759/cureus.117653

**Published:** 2026-10-08

**Authors:** Christopher Alejandro Jiménez Beltrán, Sara Camila Jimenez Beltran, Luisa Fernanda Floresvillar Slim, Fernanda Alejandra Saldívar Alcacio, Angel Antonio Aparicio Santiago, Camila Servin Figueroa, Valeria Flores, Iosune Goenaga Poupard, Lucia Viridiana Galindo Rios, Luis Carlos Murillo Lepe

**Affiliations:** 1 Psychiatry, Universidad Autónoma de Guadalajara, Guadalajara, MEX; 2 Medical Sciences, Universidad Autónoma de Guadalajara, Guadalajara, MEX; 3 Allergy and Immunology, Universidad Autónoma de Guadalajara, Guadalajara, MEX; 4 Pediatrics, Universidad Autónoma de Guadalajara, Guadalajara, MEX; 5 Internal Medicine, Universidad Autónoma de Guadalajara, Guadalajara, MEX; 6 Pharmacology, Universidad Autónoma de Guadalajara, Guadalajara, MEX; 7 Anatomy, Universidad Autónoma de Guadalajara, Guadalajara, MEX; 8 Family Medicine, Universidad Autónoma de Guadalajara, Guadalajara, MEX; 9 Gastroenterology and Hepatology, Universidad Autónoma de Guadalajara, Guadalajara, MEX

**Keywords:** antidepressant treatment, bright light therapy, chronotherapy, exercise therapy, lifestyle intervention, major depressive disorder, sleep deprivation, systematic review

## Abstract

Major depressive disorder (MDD) remains disabling despite effective pharmacotherapy, and many patients experience an incomplete response or residual symptoms. This systematic review evaluated randomized trials of structured exercise or mind-body exercise, sleep/circadian and bright-light interventions, whole-diet interventions, and multicomponent lifestyle programs delivered alongside antidepressant treatment in adults with current MDD. PubMed, Europe PMC, and ClinicalTrials.gov were searched from inception through September 15, 2026, with supplementary citation searching of eligible reports and relevant reviews. The electronic searches identified 1,310 records. After the removal of 333 duplicate bibliographic records and one non-usable record, 976 records underwent screening. Thirty-eight reports were assessed at full text. Following eligibility assessment and final verification, 26 eligible reports remained from the original search, and eight additional eligible reports were identified through supplementary citation searching. The final review included 34 reports representing 29 independent randomized studies. Risk of bias was assessed using the Cochrane Risk of Bias 2 (RoB 2) tool, certainty of evidence was evaluated using Grading of Recommendations Assessment, Development and Evaluation (GRADE), and results were synthesized without meta-analysis because substantial clinical and methodological heterogeneity made a pooled estimate inappropriate. Structured exercise showed the most consistent favorable signal, although effects were generally smaller or less certain in trials using active comparators. Sleep/circadian and bright-light interventions showed possible short-term benefits in selected studies, but findings varied by intervention, comparator, and assessment time, and sustained benefits were inconsistent. Evidence for multicomponent lifestyle programs was sparse, and no eligible randomized trial evaluated a whole-diet intervention alone as an adjunct to antidepressant treatment. Overall certainty of the evidence was low to very low because of risk of bias, inconsistency, and imprecision. Lifestyle-based interventions may provide additional benefit as adjuncts to antidepressant treatment, particularly structured exercise and selected sleep/circadian interventions; however, the magnitude and durability of benefit remain uncertain. Substitution of lifestyle interventions for antidepressant medication was not evaluated in this review.

## Introduction and background

Major depressive disorder (MDD) is a recurrent psychiatric disorder associated with impaired functioning, reduced quality of life, physical comorbidity, and suicide risk [1,2]. Antidepressant pharmacotherapy is an established component of treatment, but incomplete response, residual symptoms, adverse effects, and recurrence remain common. These limitations have increased interest in adjunctive nonpharmacologic strategies that target modifiable behavioral and physiological factors while established treatment is continued [3].

For the purposes of this review, lifestyle-based or lifestyle-related interventions were operationally defined as structured, reproducible, nonpharmacologic strategies targeting physical activity, sleep-wake or circadian processes, dietary patterns, or multiple lifestyle behaviors. Exercise and physical-activity interventions include structured aerobic, resistance, mind-body, or multimodal activity programs. Sleep and circadian interventions include behavioral manipulation of sleep-wake timing, wake therapy, therapeutic sleep deprivation, and bright light therapy. Bright light and wake therapy were retained within the sleep/circadian domain because they directly modify environmental or behavioral inputs to circadian regulation; however, they were analyzed separately from exercise, dietary, and multicomponent interventions and were not assumed to share a common mechanism or treatment effect. Multicomponent interventions were required to combine two or more structured lifestyle targets. Dietary interventions were considered only when they involved modification of an overall dietary pattern rather than isolated nutritional supplements.

Several previous reviews have addressed portions of this evidence base, but their scope differs from the present clinical question. Mura G et al. systematically reviewed exercise specifically as an add-on strategy to antidepressant treatment in MDD [4]. Penders TM et al. evaluated bright light therapy as augmentation of pharmacotherapy and reported evidence of benefit across randomized studies of nonseasonal depression [5]. More recently, Chapman H et al. reviewed a substantially broader group of nonpharmacological and nonpsychological augmentation strategies for unipolar depression [6]. Gonzalez-Garcia X et al. conducted an umbrella review of systematic reviews and meta-analyses of lifestyle interventions for adults with depression and identified exercise as one of the more consistently supported approaches [7]. These reviews provide important context but do not apply a single primary-study eligibility framework restricted to adults with current, diagnostically established MDD receiving concurrent antidepressant treatment across the lifestyle domains considered here.

The present review therefore addresses a narrower clinical question: whether structured lifestyle-related interventions provide additional benefit when used alongside antidepressant treatment in adults with current MDD. We synthesized primary randomized evidence separately for exercise or mind-body interventions, sleep/circadian and bright light interventions, whole-diet interventions, and multicomponent lifestyle programs. Particular attention was given to the randomized comparator, antidepressant-treatment context, depressive-symptom outcomes, response and remission, assessment timing, durability of benefit, and adverse events. The review was reported according to the Preferred Reporting Items for Systematic Reviews and Meta-Analyses (PRISMA) 2020 statement and the Preferred Reporting Items for Systematic Reviews and Meta-Analyses literature search extension (PRISMA-S) [8,9].

## Review

Materials and methods

Protocol and Registration

A protocol specifying the review question, eligibility criteria, information sources, study-selection procedures, data items, risk-of-bias assessment, and synthesis plan was prepared before completion of the definitive screening. The protocol was not prospectively registered in PROSPERO or another public registry. The protocol and relevant methodological details are provided in Appendix 1.

Eligibility Criteria

Eligibility criteria were structured using the Population, Intervention, Comparator, and Outcomes (PICO) framework, with study design specified as an additional eligibility domain, consistent with PRISMA 2020 [8]. Adults aged 18 years or older with current MDD established using the Diagnostic and Statistical Manual of Mental Disorders (DSM), International Classification of Diseases (ICD), or a validated diagnostic interview were eligible. Mixed diagnostic populations were included only when data for participants with MDD could be separately extracted.

Eligible interventions included structured exercise or physical activity; nonpharmacologic sleep or circadian interventions, including therapeutic sleep deprivation, wake therapy, and bright light therapy; whole-diet interventions; and multicomponent lifestyle programs. Whole-diet interventions were defined as interventions targeting an overall dietary pattern rather than isolated nutritional supplements. The intervention had to be delivered alongside antidepressant pharmacotherapy or standard psychiatric care in which antidepressant treatment was stated and comparably managed across randomized groups. Eligible comparators included the same pharmacotherapy or standard care without the index lifestyle intervention; an attention, sham, placebo, low-intensity, or behavioral control; or another eligible lifestyle intervention when the randomized comparison permitted assessment of the index intervention.

Validated depressive-symptom severity was the primary outcome. Secondary outcomes included treatment response, remission, quality of life, functioning, sleep, medication use, adherence, withdrawals, and adverse events. Randomized controlled trials were eligible. Multiple publications from the same randomized cohort were linked and counted as one independent study.

We excluded pediatric populations; bipolar, seasonal-only, postpartum, or perinatal depression when MDD-specific results were not separately extractable; studies based solely on elevated depressive-symptom scores without confirmed MDD; psychotherapy-only or pharmacological augmentation studies without an eligible lifestyle intervention; supplement-only interventions; protocols, conference abstracts, case reports, observational studies, and reports without an eligible validated depression outcome.

Information Sources and Search Strategy

PubMed, Europe PMC, and ClinicalTrials.gov were searched from database inception through September 15, 2026. No publication-year restriction was applied. PubMed and Europe PMC were retained because they were the accessible bibliographic sources used in the completed review. Their substantial overlap was anticipated, and overlapping bibliographic records were addressed during deduplication using PubMed identifier (PMID), digital object identifier (DOI), and title; the two sources were not treated as independent evidence bases.

The database-specific strategies combined terms related to MDD, exercise or physical activity, diet or nutrition, sleep-related interventions, lifestyle, antidepressant treatment, adjunctive or augmentation treatment, usual care, and randomized trials. Exact search strings varied by information source and are provided in Appendix 2.

The ClinicalTrials.gov search was restricted to completed interventional studies and returned 157 registry records. Ongoing studies were therefore not represented among these 157 records. A reliable retrospective record-level classification of all 157 records into publication-linked studies, completed but unpublished trials, and otherwise ineligible records was not retained and could not be reconstructed without unsupported assumptions. Registry entries without published outcome data were not treated as efficacy reports. Consequently, completed unpublished trials may have contributed to missing-results bias or publication bias.

Supplementary citation searching was performed by examining reference lists of eligible reports and relevant systematic reviews. Reports identified through citation searching were assessed using the same eligibility criteria as database-derived records. The complete database-specific search strategies, interfaces, search dates, yields, and supplementary citation-search procedures are provided in Appendix 2.

The search was limited to PubMed, Europe PMC, and ClinicalTrials.gov because subscription access to additional databases, including Embase, PsycINFO, and CENTRAL, was unavailable. The possibility that relevant studies were missed because of this restricted database coverage is acknowledged as a limitation.

Study Selection

Bibliographic records from PubMed and Europe PMC were normalized using PMID, DOI, and title and were deduplicated before screening. ClinicalTrials.gov registry records were retained as a separate registry set. CAJB conducted the initial title and abstract screening and full-text eligibility assessment against the prespecified criteria. For the final evidence set, SCJB independently verified the eligibility of included studies and the reasons for full-text exclusion. Disagreements were resolved by discussion and consensus between CAJB and SCJB.

All 38 reports counted as full-text assessments in the PRISMA flow were successfully obtained and were available in sufficient detail to apply the eligibility criteria. The access limitations described in this review refer to the absence of subscription access to additional bibliographic databases and, for some older studies, incomplete availability of ancillary methodological information such as protocols or prespecified analysis plans; they do not indicate that any of the 38 full-text reports were unavailable for eligibility assessment.

One principal reason for exclusion was assigned to each report excluded after full-text assessment. Full-text exclusion reasons are provided in Appendix 2. Multiple publications were linked as companion reports when they represented the same randomized cohort, based on author group, sample size, intervention, recruitment period, study characteristics, and trial-registration information.

Data Extraction

CAJB performed the primary data extraction using a standardized form. SCJB independently verified the extracted study characteristics, comparator descriptions, depression outcomes, response and remission data, follow-up, and adverse-event information against the source reports. Discrepancies were resolved by consensus.

Extracted variables included diagnostic criteria, study setting, randomized sample size, participant characteristics, antidepressant regimen, intervention characteristics and dose, duration, comparator, depression measure, response and remission, secondary outcomes, follow-up, adherence, withdrawals, and adverse events. Study-level effect estimates, 95% CIs, response and remission proportions, and other numerical results were recorded when reported by the original investigators. Values unavailable in the source report were not reconstructed or inferred. Detailed study-level extraction data are provided in Appendix 3.

Outcome and Timepoint Selection

When more than one validated depression measure was reported, the study-designated primary depression outcome was selected when clearly specified. When no primary depression outcome was explicitly designated, the validated depression measure most clearly reported across randomized groups at the end of the intervention was used. Clinician-rated and self-reported outcomes were not combined into a single effect estimate.

For the primary synthesis, the outcome assessment closest to completion of the randomized intervention was selected. Earlier assessments were retained when relevant to rapid treatment effects, particularly for sleep-deprivation and circadian interventions, and later assessments were considered separately when evaluating durability of benefit. Response and remission were kept distinct from changes in secondary outcomes such as sleep, functioning, quality of life, physical fitness, or biomarkers.

Risk-of-Bias Assessment

Risk of bias was assessed using the Cochrane Risk of Bias 2 (RoB 2) tool [10]. Judgments addressed bias arising from the randomization process, deviations from intended interventions, missing outcome data, measurement of the outcome, and selection of the reported result. RoB 2 assessments were made for the depression outcome and assessment timepoint used in the primary synthesis rather than assigning a single generic judgment to each publication.

CAJB performed the primary outcome-specific RoB 2 assessment, and SCJB independently verified the domain judgments and supporting rationales. Disagreements were resolved by consensus. Lack of participant or therapist blinding was expected for exercise and behavioral interventions and was not considered sufficient by itself to classify a trial as high risk. Judgments also considered assessor masking, outcome subjectivity, adherence, missing data, analysis approach, and selective-reporting concerns. Study-level RoB 2 judgments and supporting rationales are provided in Appendix 3.

Synthesis Method

Studies were grouped according to intervention domain: exercise or mind-body exercise, sleep/circadian or bright-light interventions, whole-diet interventions, and multicomponent lifestyle programs. Results were synthesized using structured synthesis without meta-analysis, following Synthesis Without Meta-analysis (SWiM) guidance [11]. Interpretation considered the direction and magnitude of effect, precision, comparator type and intensity, intervention dose and supervision, antidepressant-treatment context, assessment timing, durability of benefit, adherence, adverse events, and risk of bias.

Continuous and dichotomous outcomes were reported using the effect measures provided by the individual studies. Response and remission proportions and 95% CIs were reported when available.

Reason for Not Conducting a Meta-Analysis

A pooled meta-analysis was not performed because the included studies differed substantially in intervention type and dose, pharmacotherapy context, comparator intensity, depression measure, assessment timing, treatment duration, and outcome reporting. In particular, exercise studies used comparators ranging from antidepressant treatment or usual care alone to stretching, low-intensity exercise, or self-organized activity, whereas sleep and circadian studies used medication-only, regular-sleep, dim-light, sham-light, and other active comparator conditions.

Pooling these clinically different comparisons would require an assumption of exchangeability that was not considered appropriate. Accordingly, quantitative study-level findings were presented within a structured synthesis rather than as a single pooled effect. Because no meta-analysis was conducted, funnel plots and statistical tests for small-study effects were not performed.

Certainty of Evidence

Certainty of evidence was assessed using the Grading of Recommendations Assessment, Development and Evaluation (GRADE) framework [12]. Certainty was evaluated separately for clinically coherent outcome domains. Randomized evidence began at high certainty and could be downgraded for risk of bias, inconsistency, indirectness, imprecision, or publication bias.

GRADE assessments considered outcome-specific RoB 2 judgments, consistency of findings, sample size and event counts, precision of individual study estimates, comparator structure, and assessment timing. The GRADE Summary of Findings and explanations for downgrading are presented below and in Appendix 3.

Results

Study Selection

The searches identified 1,310 records: 799 from PubMed, 354 from Europe PMC, and 157 from ClinicalTrials.gov. Before screening, 334 records were removed, including 333 duplicate bibliographic records and one unusable record, leaving 976 records for initial screening. A total of 938 records were excluded during initial screening. Thirty-eight reports were retrieved and assessed for full-text eligibility. All 38 reports counted as full-text reports in the PRISMA flow were successfully obtained and assessed in sufficient detail to apply the eligibility criteria. The reported access limitations refer to the lack of subscription access to additional bibliographic databases and, for some older studies, incomplete availability of ancillary methodological information rather than failure to obtain any of these 38 full-text reports.

Nine reports were excluded for prespecified reasons. During final eligibility verification, three companion reports from a mixed unipolar/bipolar chronotherapy cohort were excluded because MDD-specific randomized outcomes were not separately extractable, leaving 26 eligible reports from the original search. Supplementary citation searching and reference-list checking identified eight additional eligible reports. The final qualitative synthesis therefore included 34 reports representing 29 independent randomized studies. The study-selection process is shown in Figure 1.

**Figure 1:**
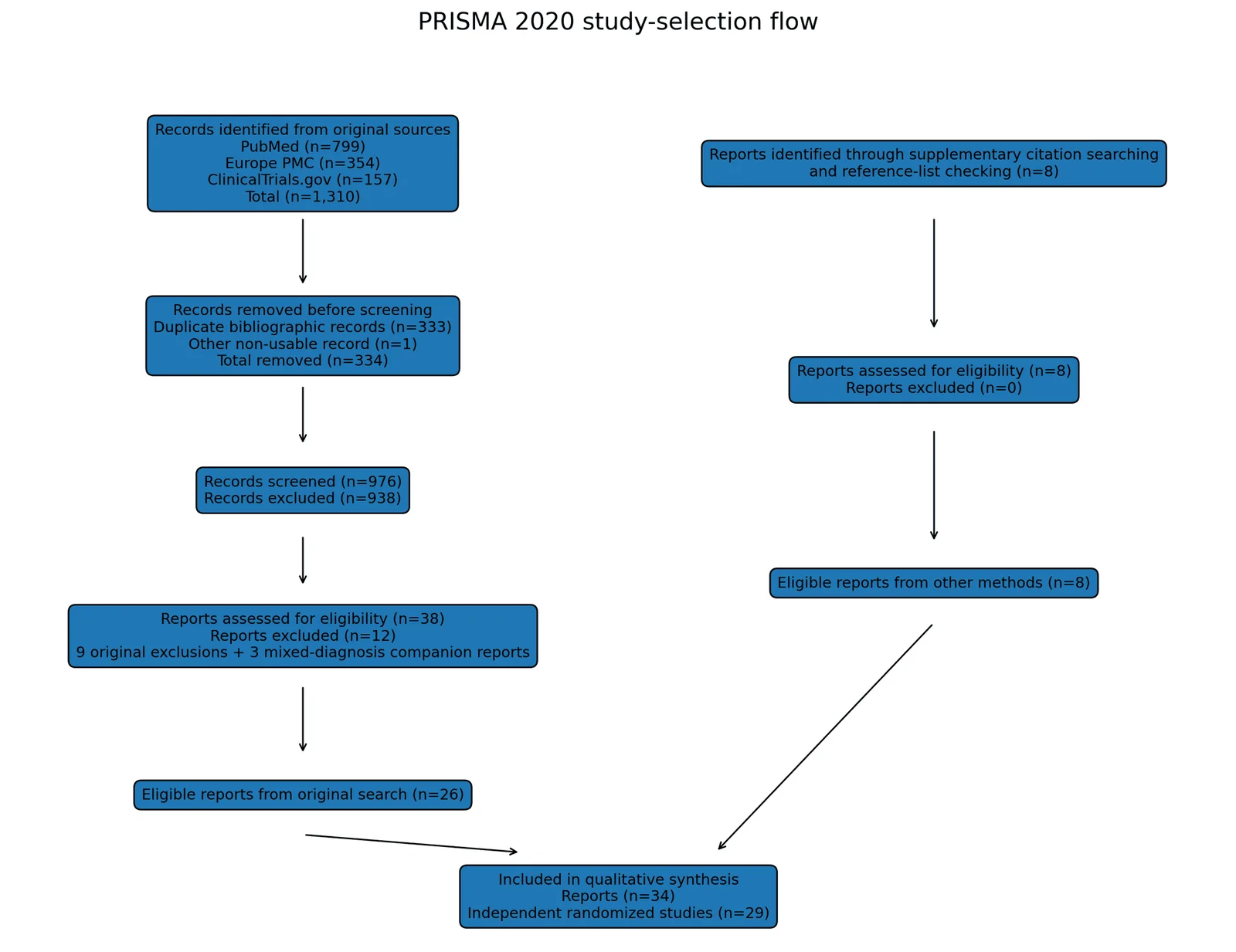
PRISMA 2020 flow diagram for study selection. PRISMA 2020 flow diagram showing records identified through PubMed, Europe PMC, and ClinicalTrials.gov; records removed before screening; records screened; reports assessed for full-text eligibility; reports excluded; reports identified through supplementary citation searching and reference-list checking; and the final inclusion of 34 reports representing 29 independent randomized studies. PRISMA: Preferred Reporting Items for Systematic Reviews and Meta-Analyses.

Study Characteristics

The final evidence set comprised 34 reports representing 29 independent randomized studies [13-46]. The 29 independent randomized studies were grouped into four prespecified intervention domains: exercise or mind-body exercise, sleep/circadian or bright-light interventions, multicomponent lifestyle programs, and whole-diet interventions. Sixteen independent randomized studies evaluated exercise or mind-body exercise [17-22,24-32,34,37-39], 11 evaluated sleep/circadian or bright-light interventions [13-16,35,36,40-46], and two evaluated multicomponent lifestyle programs [23,33]. No eligible randomized trial evaluated a whole-diet intervention alone as an adjunct to ongoing antidepressant treatment in adults with current MDD.

Sample sizes, intervention duration, treatment setting, antidepressant regimen, and comparator intensity varied considerably. Exercise comparators ranged from pharmacotherapy or usual care alone to stretching, low-intensity exercise, health education, and self-organized physical activity. Sleep/circadian trials used medication-only, regular-sleep, dim-light, sham-light, and other active comparison conditions. Companion publications arising from the same randomized cohort were linked and counted as one independent study. The individual reports contributing to the 29 independent randomized studies are summarized in Table 1.

**Table 1:** Characteristics of the included randomized studies. Companion publications from the same randomized cohort were linked and counted as one independent study. BBAT: Basic body awareness therapy; CBT: Cognitive behavioral therapy; MBCT: Mindfulness-based cognitive therapy; MDD: Major depressive disorder; SSRI: Selective serotonin reuptake inhibitor.

Study	Domain	N	Adjunct intervention	Comparator / treatment context	Duration
Kuhs H et al. (1996) [13]	Sleep/circadian	51	Amitriptyline + repeated late partial sleep deprivation	Amitriptyline alone	4 weeks
Reynolds CF 3rd et al. (2005) / Smith GS et al. (2009) [14,15]	Sleep/circadian	80; PET subset 16	Total sleep deprivation in paroxetine/placebo factorial context	Paroxetine/placebo and sleep-procedure comparison conditions	2 weeks
Güdücü F et al. (2005) [16]	Sleep/circadian	37	Sertraline + partial sleep deprivation or bright light	Sertraline alone	15 days
Pilu A et al. (2007) / Carta MG et al. (2008) [17,18]	Exercise	30	Group physical activity + ongoing antidepressants	Ongoing antidepressants alone	32 weeks
Lavretsky H et al. (2011) [19]	Mind-body exercise	73 randomized partial responders	Tai Chi Chih + escitalopram	Health education + escitalopram	10 weeks
Mota-Pereira J et al. (2011) [20]	Exercise	33	Moderate exercise + unchanged pharmacotherapy	Pharmacotherapy alone	12 weeks
Schuch FB et al. (2011) [21]	Exercise	Small inpatient pilot	Aerobic exercise + inpatient treatment	Inpatient treatment alone	Hospital stay
Trivedi MH et al. (2011) [22]	Exercise	126	Higher-dose exercise augmentation	Lower-dose exercise augmentation	12 weeks
Tanyi RA et al. (2011) [23]	Multicomponent	41	Relaxation, guided imagery, humor + CBT/pharmacotherapy	CBT/pharmacotherapy	3 weeks
Danielsson L et al. (2014) [24]	Exercise/mind-body	62	Aerobic exercise + antidepressants; BBAT active arm	Single physical-activity consultation / usual care	10 weeks
Kerling A et al. (2015) [25]	Exercise	42	Supervised exercise + inpatient treatment	Treatment as usual	6 weeks
Belvederi Murri M et al. (2015) [26]	Exercise	121	Progressive or low-intensity exercise + sertraline	Sertraline alone	24 weeks
Carneiro LS et al. (2015) [27]	Exercise	26 women	Supervised aerobic exercise + pharmacotherapy	Pharmacotherapy alone	16 weeks
Shachar-Malach T et al. (2015) [28]	Exercise	12	Aerobic exercise + antidepressants	Stretching + antidepressants	3 weeks
Legrand FD and Neff EM (2016) [29]	Exercise	35	Aerobic exercise + early antidepressant treatment	Stretching or no intervention + medication	10 days
Siqueira CC et al. (2016) [30]	Exercise	57	Aerobic exercise + sertraline	Sertraline alone	4 weeks
Moraes HS et al. (2020) [31]	Exercise	27	Aerobic or strength training + antidepressants	Low-intensity exercise + antidepressants	12 weeks
Haussleiter IS et al. (2020) [32]	Exercise	111	Guided exercise therapy + usual inpatient care	Self-organized activity + usual care	6 weeks
Garcia A et al. (2023) [33]	Multicomponent	94	Remote multimodal lifestyle program + usual treatment	MBCT or written lifestyle suggestions + usual treatment	12 months
Moreira-Neto A et al. (2024) [34]	Exercise	40	Aerobic exercise with or without motor complexity + pharmacotherapy	Non-exercise + pharmacotherapy	24 weeks
Fregna L et al. (2024) [35]	Sleep/circadian	100	Bright light + stable SSRI regimen	Stable SSRI regimen alone	Acute inpatient course
Xu YH et al. (2024) [36]	Sleep/circadian	54	36-hour therapeutic sleep deprivation + medication	Regular sleep + medication	7 days
Tavares VD et al. (2025) / Barros ML et al. (2025) [37-39]	Exercise	59 in primary efficacy comparison	Exercise + pharmacotherapy	Pharmacotherapy alone	12 weeks + follow-up
Martiny K et al. (2005) [40,41]	Sleep/circadian	102	10,000-lux bright light + sertraline 50 mg	Dim red light + sertraline 50 mg	5 weeks
Prasko J et al. (2002) [42]	Sleep/circadian	34	Bright light and/or imipramine	Dim red light and/or imipramine comparator arms	3 weeks
Loving RT et al. (2002) [43]	Sleep/circadian	13 completers	Wake treatment followed by bright light + antidepressants	Wake treatment followed by dim red light + antidepressants	1 week
Güzel Özdemir P et al. (2015) [44]	Sleep/circadian	50	Venlafaxine 150 mg + bright light	Venlafaxine 150 mg alone	8 weeks
Lam RW et al. (2016) [45]	Sleep/circadian	122 total	Bright light + fluoxetine 20 mg	Sham device + fluoxetine 20 mg	8 weeks
Chen IP et al. (2022) [46]	Sleep/circadian	43	Bright light + stable antidepressant regimen	Dim red placebo light + stable antidepressant regimen	4 weeks

Quantitative Study-Level Findings

Study-level numerical results were extracted whenever available, including depressive-symptom outcomes, response and remission proportions, effect estimates, p-values, follow-up duration, withdrawals, and adverse events. The principal quantitative findings are summarized in Table 2. Values unavailable in the original reports were not reconstructed or inferred.

**Table 2:** Principal depression outcomes and quantitative findings. Numerical results are reported as provided in the source publications. Values not reported by the original investigators were not reconstructed. AE: adverse events; BDI: Beck Depression Inventory; BDI-II: Beck Depression Inventory-II; DASS-21: Depression Anxiety Stress Scales-21; ECT: Electroconvulsive therapy; HAM-D/HDRS: Hamilton Depression Rating Scale; HAM-D6: 6-item Hamilton Depression Rating Scale; HAM-D17: 17-item Hamilton Depression Rating Scale; HAM-D24: 24-item Hamilton Depression Rating Scale; HDRS-17: 17-item Hamilton Depression Rating Scale; IDS-C: Inventory of Depressive Symptomatology, Clinician-Rated; MADRS: Montgomery-Åsberg Depression Rating Scale; NNT: Number needed to treat; SIGH-SAD: Structured Interview Guide for the Hamilton Depression Rating Scale-Seasonal Affective Disorder Version.

Study	Primary outcome / timepoint	Principal quantitative result	Assessment / follow-up	Adverse events / attrition
Kuhs H et al. (1996) [13]	HAM-D, week 4	Observer-rated improvement/response favored adjunct sleep deprivation; self-rating did not confirm superiority.	Not reported	AE detail incomplete
Reynolds CF 3rd et al. (2005) / Smith GS et al. (2008) [14,15]	HAM-D, week 2	No sustained acceleration or symptomatic superiority.	2 weeks	AE detail incomplete
Güdücü F et al. (2005) [16]	HAM-D, day 15	Sleep-deprivation arm improved earlier; adjunct arms also improved anxiety.	15 days	Sparse AE reporting
Pilu A et al. (2007) / Carta MG et al. (2008) [17,18]	Clinical depression outcomes, month 8	Adjunct activity favored clinical/functional outcomes; a precise between-group CI was not available for extraction.	32 weeks	Attrition/reporting incomplete
Lavretsky H et al. (2011) [19]	HAM-D/remission, week 10	Greater symptom reduction and remission with Tai Chi Chih + escitalopram.	10 weeks	No major safety signal highlighted
Mota-Pereira J et al. (2011) [20]	HAM-D/BDI, week 12	Response 21% and remission 26% vs. 0%/0%; continuous outcomes favored exercise.	12 weeks	AE reporting sparse
Schuch FB et al. (2011) [21]	HAM-D at discharge	Week 2: 8.2 vs. 11.18 (p = .192); discharge: 5.93 vs. 9.45 (p = .041). ECT imbalance limits attribution.	Discharge	No robust AE comparison
Trivedi MH et al. (2011) [22]	IDS-C remission, week 12	Adjusted remission 28.3% vs. 15.5%; NNT 7.8; p = .07 between doses.	12 weeks	Adherence/dropout relevant
Tanyi RA et al. (2011) [23]	Depression scale, week 3	Within-group improvement; between-group precision/component attribution limited.	3 weeks	AE detail incomplete
Danielsson L et al. (2014) [24]	MADRS, week 10	Reported mean change −10.3 points (95% CI, −13.5 to −7.1); missing data/expectations limit inference.	10 weeks	No major safety signal highlighted
Kerling A et al. (2015) [25]	MADRS/response, week 6	Response was more frequent with exercise; exact event counts were not available for extraction.	6 weeks	AE detail incomplete
Belvederi Murri M et al. (2015) [26]	HAM-D remission, week 24	Remission 81% progressive, 73% low-intensity, 45% sertraline; p = .001.	24 weeks	Complete comparative AE data were not available for extraction
Carneiro LS et al. (2015) [27]	BDI-II/DASS-21, week 16	Depressive symptoms and fitness favored exercise; small sample limited precision.	16 weeks	Small sample
Shachar-Malach T et al. (2015) [28]	HAM-D response/remission, week 3	Response 4/6 vs. 0/6; remission 2/6 vs. 0/6.	3 weeks	Very small sample
Legrand FD and Neff EM (2016) [29]	BDI-II, day 10	Aerobic vs. stretching Cohen's d = −1.06; no significant improvement in the no-intervention control.	10 days	Short follow-up
Siqueira CC et al. (2016) [30]	HAM-D, week 4	Both groups improved; the exercise group used a lower sertraline dose and showed improved fitness.	4 weeks	Detailed AE counts incomplete
Moraes HS et al. (2020) [31]	HAM-D/BDI, week 12	Aerobic HAM-D response vs. control p = .044; strength-training BDI response vs. control p = .044.	12 weeks	Small sample
Haussleiter IS et al. (2020) [32]	HAM-D, week 6	Guided exercise reduced HAM-D more than self-organized activity (p = .017).	6 weeks	Detailed comparative AE counts were not available for extraction
Garcia A et al. (2023) [33]	BDI-II, month 12	No significant between-group BDI-II benefit; selected secondary lifestyle and quality-of-life outcomes improved.	12 months	Long follow-up; attrition relevant
Moreira-Neto A et al. (2024) [34]	Depression/affective outcomes, week 24	Hopelessness/affect favored exercise; depression-severity contrast was not the principal efficacy result.	24 weeks	Small pilot
Fregna L et al. (2024) [35]	HAM-D, acute endpoint	Depression improved in both groups; subjective sleep quality improved more clearly with bright light.	Acute course	Comparative AE estimate not precise
Xu YH et al. (2024) [36]	HAM-D24, day 7	Day-3 response 34.8% vs. 3.2%; no sustained day-7 difference.	7 days	Supervised setting; missing data relevant
Tavares VD et al. / Barros ML et al. (2025) [37-39]	Depression/remission, week 12	Remission 42.3% vs. 27.2% initially and 72.7% vs. 48.1% later; benefit was maintained during follow-up.	4 months	Exercise dropout 15.3%; AE reporting available in source
Martiny K et al. (2005) [40,41]	HAM-D6/HAM-D17, week 5	Clinician-rated depression reduction was significantly greater with bright light + sertraline.	5 weeks	Double-blind placebo-light design
Prasko J et al. (2002) [42]	HAM-D/SIGH-SAD, week 3	All arms improved; between-group differences were not statistically significant.	3 weeks	Small sample
Loving RT et al. (2002) [43]	HDRS-17, week 1	Bright-light arm improved 27%; between-group significance depended on exclusion of one placebo outlier.	1 week	Very small sample
Güzel Özdemir P et al. (2015) [44]	HDRS, week 8	Week 4 HDRS ≤13: 76% vs. 44%; week 8 remission 76% vs. 64% (p = .36).	8 weeks	No sham-light control
Lam RW et al. (2016) [45]	MADRS, week 8	Mean change 16.9 vs. 8.8; p = .02. Response 75.9% vs. 29.0%; remission 58.6% vs. 19.4%.	8 weeks	Generally well tolerated
Chen IP et al. (2022) [46]	HAM-D17, week 4	No group-by-time effect; response 31.8% vs. 33.3%; remission 13.6% vs. 23.8%.	4 weeks	Small sample

Exercise and Mind-Body Interventions

Exercise and mind-body interventions constituted the largest evidence domain. Several trials comparing structured exercise with pharmacotherapy or usual care alone reported greater reductions in depressive symptoms or higher response or remission rates with exercise augmentation. However, effects were less consistent when the comparator itself contained structured physical activity, stretching, health education, or behavioral engagement [17-22,24-32,34,37-39].

In the TREAD trial, higher-dose exercise augmentation produced an adjusted remission rate of 28.3% compared with 15.5% with lower-dose exercise, corresponding to a reported number needed to treat of 7.8; the group effect did not reach conventional statistical significance (p = 0.07) [22]. Belvederi MM et al. reported remission rates of 81% with progressive aerobic exercise, 73% with lower-intensity exercise, and 45% with sertraline alone at 24 weeks (p = 0.001) [26]. Shachar-Malach T et al. reported response in four of six participants receiving aerobic exercise compared with none of six receiving stretching; remission occurred in two of six versus none, respectively [28]. The very small sample substantially limits precision.

Legrand and Neff reported a large short-term effect favoring aerobic exercise over stretching on Beck Depression Inventory-II (BDI-II) scores (Cohen's d = -1.06) during the initial phase of antidepressant treatment [29]. Haussleiter IS et al. found a greater reduction in Hamilton Depression Rating Scale (HAM-D) scores with guided exercise than with self-organized physical activity during inpatient treatment (p = 0.017) [32]. The Tavares randomized cohort also reported higher remission proportions with adjunctive exercise during treatment and follow-up [37-39]. Taken together, exercise results generally favored structured intervention, but the magnitude and consistency of benefit depended substantially on comparator intensity, sample size, treatment setting, and study quality.

Sleep/Circadian and Bright-Light Interventions

Eleven independent studies evaluated sleep deprivation, wake therapy, bright light therapy, or related circadian interventions [13-16,35,36,40-46]. Several trials suggested rapid antidepressant effects, particularly during the first days of treatment, but these effects were not consistently sustained. Xu YH et al. reported a response rate of 34.8% on day 3 following 36-hour therapeutic sleep deprivation plus medication compared with 3.2% with regular sleep plus medication. The advantage was not sustained at day 7 [36].

Bright-light trials produced both positive and neutral findings. Martiny K et al. randomized 102 participants receiving sertraline to bright light or dim red light and reported greater clinician-rated improvement with bright light [40,41]. Prasko J et al. found improvement across treatment groups but no statistically significant between-group difference [42]. Loving RT et al. reported approximately 27% improvement in Hamilton Depression Rating Scale (HDRS) scores in the bright-light condition, although the between-group inference was sensitive to one placebo-group outlier [43].

Güzel ÖP et al. reported an HDRS score of 13 or lower at week 4 in 76% of participants receiving venlafaxine plus bright light compared with 44% receiving venlafaxine alone. At week 8, remission was 76% versus 64%, respectively, and the difference was not statistically significant (p = 0.36) [44]. Lam RW et al. reported greater Montgomery-Åsberg Depression Rating Scale (MADRS) improvement with bright light plus fluoxetine than with sham treatment plus fluoxetine. Mean improvement was 16.9 versus 8.8 points (p = 0.02), with response rates of 75.9% versus 29.0% and remission rates of 58.6% versus 19.4% [45]. In contrast, Chen IP et al. found no significant group-by-time effect after four weeks of treatment; response rates were 31.8% versus 33.3%, and remission rates were 13.6% versus 23.8% for bright light and placebo light, respectively [46].

Overall, sleep/circadian and bright-light interventions showed potential short-term benefit in some studies, but findings differed by intervention, comparator, and assessment time, and sustained benefit was less consistent.

Multicomponent Lifestyle and Whole-Diet Interventions

Only two randomized studies evaluated multicomponent lifestyle programs [23,33]. The interventions differed considerably in content and treatment setting. The larger study involving patients with treatment-resistant depression did not demonstrate a significant between-group improvement in BDI-II depressive symptoms, although improvements were reported for selected secondary lifestyle and quality-of-life outcomes [33].

No eligible randomized trial evaluated a whole-diet intervention alone as an adjunct to ongoing antidepressant treatment in adults with current MDD. This finding reflects the narrow eligibility criteria of the review and should not be interpreted as evidence that dietary interventions are ineffective for depression.

Risk of Bias

Risk-of-bias judgments were made for the depression outcome and assessment timepoint used in the primary synthesis. Common concerns included incomplete reporting of allocation procedures in older trials, missing outcome data, reliance on subjective outcomes, lack of masked outcome assessment in some studies, cointerventions, small samples, and limited availability of prespecified analysis plans. Because participant and therapist masking is generally not feasible for exercise and behavioral interventions, the absence of such masking was not considered sufficient by itself to classify a study as high risk. The outcome-specific RoB 2 judgments are shown in Table 3.

**Table 3:** Outcome-specific Cochrane RoB 2 judgments for the primary depression outcome. Outcome- and timepoint-specific Cochrane Risk of Bias 2 (RoB 2) judgments for the 29 independent randomized studies [13-46]. D1: Bias arising from the randomization process; D2: Bias due to deviations from intended interventions; D3: Bias due to missing outcome data; D4: Bias in measurement of the outcome; D5: Bias in selection of the reported result. Overall judgments were classified as low risk of bias, some concerns, or high risk of bias [10]. BDI: Beck Depression Inventory; BDI-II: Beck Depression Inventory-II; DASS-21: Depression Anxiety Stress Scales-21; HAM-D/HAMD/HDRS: Hamilton Depression Rating Scale; HAM-D6: 6-item Hamilton Depression Rating Scale; HAM-D17/HAMD-17/HDRS-17: 17-item Hamilton Depression Rating Scale; HAM-D24: 24-item Hamilton Depression Rating Scale; IDS-C: Inventory of Depressive Symptomatology, Clinician-Rated; MADRS: Montgomery-Åsberg Depression Rating Scale; RoB 2: Risk of Bias 2; SIGH-SAD: Structured Interview Guide for the Hamilton Depression Rating Scale–Seasonal Affective Disorder Version.

Study	Outcome / timepoint	D1	D2	D3	D4	D5	Overall
Kuhs H et al. (1996) [13]	HAM-D, week 4	Some concerns	Some concerns	Low	Some concerns	Some concerns	Some concerns
Reynolds CF 3rd et al. (2005) / Smith GS et al. (2009) [14,15]	HAM-D, week 2	Low	Low	Some concerns	Low	Some concerns	Some concerns
Güdücü F et al. (2005) [16]	HAM-D, day 15	Some concerns	Some concerns	Some concerns	Some concerns	Some concerns	Some concerns
Pilu A et al. (2007) / Carta MG et al. (2008) [17,18]	Clinical depression, month 8	Some concerns	High	High	Some concerns	High	High
Lavretsky H et al. (2011) [19]	HAM-D/remission, week 10	Low	Some concerns	Some concerns	Low	Some concerns	Some concerns
Mota-Pereira J et al. (2011) [20]	HAM-D/BDI, week 12	Some concerns	Some concerns	Some concerns	Some concerns	Some concerns	Some concerns
Schuch FB et al. (2011) [21]	HAM-D, discharge	Some concerns	High	Some concerns	Low	Some concerns	High
Trivedi MH et al. (2011) [22]	IDS-C remission, week 12	Low	Some concerns	Some concerns	Low	Low	Some concerns
Tanyi RA et al. (2011) [23]	Depression scale, week 3	Some concerns	High	Some concerns	High	Some concerns	High
Danielsson L et al. (2014) [24]	MADRS, week 10	Low	Some concerns	Some concerns	Low	Some concerns	Some concerns
Kerling A et al. (2015) [25]	MADRS/response, week 6	Some concerns	Some concerns	Some concerns	Low	Some concerns	Some concerns
Belvederi Murri M et al. (2015) [26]	HAM-D remission, week 24	Low	Some concerns	Some concerns	Low	Low	Some concerns
Carneiro LS et al. (2015) [27]	BDI-II/DASS-21, week 16	Some concerns	Some concerns	Some concerns	High	Some concerns	High
Shachar-Malach T et al. (2015) [28]	HAM-D response/remission, week 3	Some concerns	Some concerns	High	Low	High	High
Legrand FD and Neff EM (2016) [29]	BDI-II, day 10	Some concerns	Some concerns	Some concerns	High	Some concerns	High
Siqueira CC et al. (2016) [30]	HAM-D, week 4	Low	Some concerns	Low	Low	Low	Some concerns
Moraes HS et al. (2020) [31]	HAM-D/BDI, week 12	Some concerns	Some concerns	Some concerns	Low	Some concerns	Some concerns
Haussleiter IS et al. (2020) [32]	HAM-D, week 6	Some concerns	Some concerns	Some concerns	Low	Some concerns	Some concerns
Garcia A et al. (2023) [33]	BDI-II, month 12	Low	Some concerns	Some concerns	High	Low	High
Moreira-Neto A et al. (2024) [34]	Depression/affective, week 24	Some concerns	Some concerns	Some concerns	High	Some concerns	High
Fregna L et al. (2024) [35]	HAM-D, acute endpoint	Some concerns	Some concerns	Some concerns	Low	Some concerns	Some concerns
Xu YH et al. (2024) [36]	HAM-D24, day 7	Low	Some concerns	High	Low	Low	High
Tavares VD et al. (2025) / Barros ML et al. (2025) [37-39]	Depression/remission, week 12	Low	Some concerns	Some concerns	Some concerns	Some concerns	Some concerns
Martiny K et al. (2005) [40,41]	HAM-D6/HAM-D17, week 5	Low	Low	Some concerns	Low	Some concerns	Some concerns
Prasko J et al. (2002) [42]	HAM-D/SIGH-SAD, week 3	Some concerns	Some concerns	Some concerns	Some concerns	Some concerns	Some concerns
Loving RT et al. (2002) [43]	HDRS-17, week 1	Some concerns	Some concerns	Some concerns	Some concerns	High	High
Güzel Özdemir P et al. (2015) [44]	HDRS, week 8	Some concerns	Some concerns	Low	Some concerns	Some concerns	Some concerns
Lam RW et al. (2016) [45]	MADRS, week 8	Low	Low	Some concerns	Low	Low	Some concerns
Chen IP et al. (2022) [46]	HAMD-17, week 4	Low	Low	Some concerns	Low	Some concerns	Some concerns

Certainty of Evidence

Formal GRADE assessment was conducted for clinically coherent outcome domains. Certainty for exercise-related depressive-symptom severity was rated low, principally because of risk of bias and inconsistency across comparator types and settings. Certainty for exercise-related response and remission was also rated low, primarily because of risk of bias and imprecision.

Certainty for sleep/circadian and bright-light depression outcomes was rated very low because positive and neutral findings coexisted, interventions and assessment times differed substantially, several trials were small or short, and risk-of-bias concerns remained. Certainty for multicomponent lifestyle interventions was rated very low because only two heterogeneous studies were available and estimates were imprecise. Safety and acceptability evidence was rated very low because adverse events were incompletely and inconsistently reported. No GRADE rating was assigned to whole-diet-only adjunctive treatment because no eligible randomized trial was identified. The GRADE Summary of Findings is presented in Table 4.

**Table 4:** GRADE summary of findings. GRADE: Grading of Recommendations Assessment, Development and Evaluation; RCT: Randomized controlled trial. Randomized evidence was initially rated as high certainty and was downgraded for risk of bias, inconsistency, indirectness, imprecision, or publication bias [12]. Evidence counts are outcome-specific and may therefore be lower than the total number of studies within an intervention domain, as not every randomized study contributed data to every assessed outcome. Publication bias was considered uncertain because formal assessment of small-study effects was not feasible, and completed but unpublished trials identified in trial registries could not be reliably classified retrospectively.

Outcome/domain	Evidence	Risk of bias	Inconsistency	Indirectness	Imprecision	Publication bias	Certainty	Interpretation
Exercise/mind-body: depressive symptom severity	15 RCT clusters	Serious	Serious	Not serious	Not serious	Uncertain	Low	Most trials favored structured exercise, although findings from active-control and pragmatic comparisons were less consistent.
Exercise: response/remission	10 RCT clusters	Serious	Not serious	Not serious	Serious	Uncertain	Low	Several estimates were favorable; however, small event counts and imprecision limit confidence in the findings.
Sleep/circadian/bright-light interventions: depression outcomes	11 RCT clusters	Serious	Serious	Not serious	Serious	Uncertain	Very low	Positive and neutral findings coexist; intervention modality and timing varied, and many studies were small or of short duration.
Multicomponent lifestyle interventions: depression severity	2 RCT clusters	Serious	Serious	Not serious	Serious	Uncertain	Very low	Two heterogeneous studies provide inconsistent and imprecise evidence.
Adverse events/acceptability	Across 29 RCT clusters	Serious	Not serious	Serious	Serious	Uncertain	Very low	Reporting of adverse events and acceptability was incomplete and inconsistent.
Whole-diet-only adjunctive interventions	0 eligible RCTs	-	-	-	-	-	Not assessable	No eligible randomized trials were identified.

Discussion

Interpretation of Findings

This review indicates that lifestyle-related interventions may provide additional benefit when used alongside antidepressant treatment in adults with current MDD, although the certainty and magnitude of benefit vary substantially by intervention domain and comparator.

Exercise had the largest evidence base. Trials comparing structured exercise with medication or usual care alone generally reported larger improvements than trials using active comparators such as stretching, low-intensity exercise, health education, or self-organized physical activity [17-22,24-32,34,37-39]. This pattern suggests that comparator intensity is an important determinant of the observed between-group effect. Supervision, intervention dose, adherence, patient characteristics, and clinical setting may also contribute to differences across exercise trials. Structured and supervised programs frequently produced favorable results; however, the available studies were not designed to establish supervision itself as an independent causal moderator.

Small positive trials should be interpreted cautiously. Several exercise studies enrolled fewer than 50 participants, and some of the largest apparent treatment effects were based on very small event counts. Larger active-control or pragmatic trials generally produced more modest or less certain effects.

Sleep and circadian interventions differed from exercise in their time course. Therapeutic sleep deprivation and wake therapy sometimes produced rapid improvement within days, but this benefit was not consistently maintained [13-16,35,36,40-46]. Rapid symptom reduction should therefore be distinguished from end-of-treatment response, remission, or sustained benefit.

Bright-light therapy produced mixed results. Some well-defined adjunctive comparisons showed clinically meaningful improvement, including the Martiny and Lam trials [40,41,45], whereas Prasko and Chen did not demonstrate clear superiority [42,46]. These differences may reflect variation in light intensity, timing, comparator credibility, antidepressant regimen, duration, and study population.

Comparison With Previous Reviews

The findings are broadly consistent with previous reviews while addressing a narrower clinical question. Mura G et al. evaluated exercise specifically as an add-on to antidepressant treatment and reported adjunctive benefit while emphasizing methodological limitations [4]. The present review similarly identifies a potential exercise benefit but demonstrates that estimates are generally less pronounced when exercise is compared with an active behavioral or physical-activity control.

Penders TM et al. reported a positive overall signal for bright-light augmentation of pharmacotherapy [5]. The present MDD-specific synthesis is more cautious because the eligible evidence includes both positive and neutral randomized findings.

Chapman H et al. evaluated a substantially broader range of nonpharmacological and nonpsychological augmentation strategies for depression [6]. In contrast, the present review focuses specifically on lifestyle-related interventions used alongside antidepressant treatment in diagnostically established current MDD.

Gonzalez-Garcia X et al. synthesized systematic reviews and meta-analyses of lifestyle interventions for depression and identified exercise as one of the more consistently supported approaches [7]. Differences between their conclusions and those of the present review are expected because their umbrella-review evidence base incorporated broader populations and intervention contexts.

The absence of eligible whole-diet-only adjunctive trials in the present review therefore should not be interpreted as evidence against dietary treatment. Rather, it reflects the specific requirement that participants have current MDD and receive concurrently managed antidepressant treatment.

Clinical Implications and Research Priorities

The available evidence supports considering structured exercise as a possible adjunct within comprehensive MDD treatment, while recognizing that certainty remains low and the magnitude of benefit is uncertain.

Selected sleep/circadian interventions may also have a role in supervised clinical settings, particularly when rapid symptom improvement is a treatment goal. Therapeutic sleep deprivation and wake therapy require appropriate monitoring because fatigue, relapse after recovery sleep, mood switching, and underlying suicide risk may require clinical attention. Bright-light therapy may provide adjunctive benefit in some patients, but the mixed randomized evidence does not establish a uniform treatment effect across settings and protocols.

Secondary improvements in sleep, physical fitness, quality of life, inflammatory biomarkers, or medication use should not be interpreted as equivalent to antidepressant response or remission unless validated depression outcomes also demonstrate benefit.

Future randomized trials should use adequate sample sizes, prospective registration, transparent allocation procedures, consistent antidepressant management across treatment arms, clearly defined primary depression outcomes, appropriate active comparators, complete response and remission reporting, intervention-fidelity and adherence measures, systematic adverse-event assessment, and longer follow-up. Randomized whole-diet interventions specifically designed as adjuncts to antidepressant pharmacotherapy in adults with confirmed current MDD remain an important evidence gap.

Strengths and Limitations

Strengths of this review include its diagnosis-specific adjunctive clinical question, explicit antidepressant-treatment requirement, searches from database inception, linkage of companion publications from overlapping randomized cohorts, separation of clinically distinct lifestyle domains, quantitative study-level reporting, outcome-specific RoB 2 assessment, and formal GRADE evaluation. The review also distinguishes active from inactive comparators and separates rapid treatment effects from end-of-treatment and longer-term outcomes.

Several limitations should be considered. The electronic search was restricted to PubMed, Europe PMC, and ClinicalTrials.gov and did not include Embase, PsycINFO, or CENTRAL. Supplementary citation searching reduced but does not eliminate the possibility that eligible studies were missed.

The ClinicalTrials.gov search identified 157 completed interventional registry records. A reliable retrospective record-level classification of all registry records into publication-linked studies, completed but unpublished trials, and otherwise ineligible records was not retained and could not be reconstructed without unsupported assumptions. Because registry entries without published outcome data were not treated as efficacy reports, completed unpublished trials may have contributed to missing-results bias or publication bias.

The initial screening and extraction were performed by CAJB, with final eligibility, extracted data, and RoB 2 judgments independently verified by SCJB. Although this provides an additional verification step, it is not equivalent to fully independent duplicate screening from the start of the review process.

Many included studies had small samples, short treatment periods, heterogeneous comparators, incomplete adverse-event reporting, or methodological limitations. These factors reduce precision and certainty. Heterogeneity in intervention type, dose, comparator, antidepressant regimen, outcome measure, and assessment timing precluded a clinically coherent pooled estimate. Consequently, publication bias could not be evaluated using funnel plots or formal small-study-effect tests.

Finally, synthesis without meta-analysis necessarily involves judgment when comparing heterogeneous estimates. This limitation was addressed by providing study-level numerical outcomes, distinguishing comparator structures and assessment timepoints, and integrating outcome-specific risk-of-bias and GRADE assessments.

## Conclusions

Lifestyle-based interventions may provide additional benefit when used alongside antidepressant treatment in adults with MDD, but the overall certainty of the evidence is low to very low, and treatment effects vary across intervention type, comparator, and assessment timepoint. Structured exercise showed the most consistent favorable signal, although effect sizes were generally smaller or less certain in trials using active comparators. Sleep and circadian interventions, including therapeutic sleep deprivation, wake therapy, and bright light therapy, showed possible short-term benefits in selected settings, but findings were heterogeneous and durability was inconsistent. Evidence for multicomponent lifestyle programs was limited, and no eligible randomized trial evaluated a whole-diet intervention alone as an adjunct to antidepressant treatment.

These findings concern the adjunctive use of lifestyle interventions alongside antidepressant treatment. This review did not evaluate the substitution of lifestyle interventions for antidepressant medication; therefore, no conclusion can be drawn regarding medication replacement. Larger, preregistered randomized trials with appropriate active comparators, standardized antidepressant management, prespecified depression outcomes, longer follow-up, and complete adverse-event reporting are needed to define the magnitude, durability, and clinical relevance of these adjunctive strategies.

## Appendices

Appendix 1

Review Protocol and Amendments

Review question: Among adults with a current diagnosis of major depressive disorder, what benefits and harms are associated with structured lifestyle-based interventions delivered alongside antidepressant pharmacotherapy or standard psychiatric care in which antidepressant treatment is concurrently maintained?

Objective: To identify and synthesize randomized evidence evaluating lifestyle-related interventions used as adjuncts to antidepressant treatment in adults with current major depressive disorder, with particular attention to depressive-symptom severity, response, remission, quality of life, functioning, sleep, adherence, medication use, and adverse events.

Population: Adults aged 18 years or older with current major depressive disorder established using DSM, ICD, or a validated diagnostic interview. Mixed diagnostic populations were eligible only when MDD-specific randomized data could be separately extracted.

Interventions:

Structured exercise or physical activity. Mind-body exercise. Behavioral sleep or circadian interventions. Therapeutic sleep deprivation or wake therapy. Bright light therapy. Whole-diet interventions targeting an overall dietary pattern. Multicomponent lifestyle programs combining at least two structured lifestyle targets. Isolated nutritional supplements were not considered whole-diet interventions.

Comparator: The same antidepressant pharmacotherapy or standard psychiatric care without the index lifestyle intervention. Attention, sham, placebo, low-intensity, or behavioral control conditions. Another eligible lifestyle intervention when the randomized comparison permitted assessment of the index intervention.

Outcomes: The primary outcome was validated depressive-symptom severity. Secondary outcomes included treatment response, remission, quality of life, functioning, sleep outcomes, medication use, treatment adherence, withdrawals, and adverse events.

For the primary synthesis, the depression assessment closest to the end of the randomized intervention was selected. Earlier assessments were retained when clinically relevant to rapid effects, particularly in sleep-deprivation and circadian studies, and later assessments were considered separately when evaluating durability.

Study design: Randomized controlled trials constituted the primary eligible study design. Companion reports arising from the same randomized cohort were linked and counted as one independent study.

Protocol chronology: Protocol version 1.1 was dated September 15, 2026. The protocol specified the MDD population, adjunctive antidepressant-treatment context, lifestyle intervention domains, randomized comparator requirement, primary depression outcome, RoB 2 assessment, and GRADE evaluation.

The protocol was not prospectively registered in PROSPERO or another public registry.

The completed electronic search was limited to PubMed, Europe PMC, and ClinicalTrials.gov because subscription access to additional bibliographic databases was unavailable.

CAJB completed the initial screening, primary data extraction, and RoB 2 assessment. SCJB independently verified final eligibility decisions, reasons for full-text exclusions, extracted data, and outcome-specific RoB 2 judgments.

**Table 5:** Protocol chronology and methodological amendments. RoB: Risk of Bias; RoB 2: Cochrane Risk of Bias 2 tool.

Area	Protocol intent	Final method	Rationale
Information sources	Broader subscription-database coverage was planned	The completed original search used PubMed, Europe PMC, and ClinicalTrials.gov; substantial overlap between PubMed and Europe PMC was addressed during deduplication.	These were the accessible information sources available to the review team. Subscription access to additional databases was unavailable, and restricted database coverage is explicitly acknowledged as a limitation.
Selection, extraction, and RoB	Independent duplicate processes were planned	CAJB completed the initial screening and primary extraction/RoB 2 assessment; SCJB independently verified final eligibility, full-text exclusions, extracted data, and outcome-specific RoB 2 judgments.	Final independent verification was used to reduce selection, extraction, and appraisal errors; the initial screening was not retrospectively described as duplicate screening.
Supplementary citation searching	Reference-list checking was permitted	Reference lists of eligible reports and relevant systematic reviews were checked using the same eligibility criteria; eight additional eligible reports were identified.	Reports identified through other methods were kept distinct from records identified through the original electronic searches.

Appendix 2

Complete Search Strategies and Study-Selection Documentation

PubMed search: ("major depressive disorder"[tiab] OR "major depression"[tiab]) AND ("Exercise"[Mesh] OR "Exercise Therapy"[Mesh] OR exercis*[tiab] OR "physical activity"[tiab] OR diet*[tiab] OR nutrition*[tiab] OR "Mediterranean diet"[tiab] OR sleep[tiab] OR insomnia[tiab] OR lifestyle[tiab]) AND (antidepressant*[tiab] OR "Antidepressive Agents"[Mesh] OR pharmacotherap*[tiab] OR medication*[tiab] OR "treatment as usual"[tiab] OR "usual care"[tiab] OR adjunct*[tiab] OR add-on[tiab]) AND (randomized controlled trial[pt] OR controlled clinical trial[pt] OR random*[tiab]) NOT (animals[mh] NOT humans[mh])

Interface: PubMed Search date: September 15, 2026 Yield: 799 records

Europe PMC search: (TITLE_ABS:"major depressive disorder" OR TITLE_ABS:"major depression") AND (TITLE_ABS:exercise OR TITLE_ABS:"physical activity" OR TITLE_ABS:diet OR TITLE_ABS:sleep OR TITLE_ABS:lifestyle) AND (TITLE_ABS:antidepressant OR TITLE_ABS:adjunctive OR TITLE_ABS:augmentation OR TITLE_ABS:"treatment as usual") AND (TITLE_ABS:randomized OR TITLE_ABS:randomised OR PUB_TYPE:"randomized controlled trial")

Interface: Europe PMC REST search Search date: September 15, 2026 Yield: 354 records

ClinicalTrials.gov search: AREA[ConditionSearch]("Major Depressive Disorder") AND AREA[InterventionSearch](exercise OR diet OR sleep OR lifestyle) AND AREA[OverallStatus]COMPLETED AND AREA[StudyType]INTERVENTIONAL

Interface: ClinicalTrials.gov API Search date: September 15, 2026 Yield: 157 registry records

Registry-record classification note: The ClinicalTrials.gov strategy was restricted to completed interventional studies; ongoing studies were therefore not represented among the 157 records. A reliable retrospective classification of all 157 records into publication-linked studies, completed but unpublished trials, and otherwise ineligible records was not retained and could not be reconstructed without unsupported assumptions. Registry records without published outcome data were not treated as efficacy reports. Completed but unpublished trials therefore remain a potential source of missing-results or publication bias.

Supplementary citation searching: Reference lists of eligible reports and relevant systematic reviews were examined for potentially eligible randomized trials. Reports identified through this method were assessed using the same population, intervention, comparator, outcome, and study-design criteria applied to electronically retrieved records.

Eight additional eligible reports were identified through supplementary citation searching and reference-list checking.

**Table 6:** Original study-selection arithmetic.

Selection item	n
PubMed	799
Europe PMC	354
ClinicalTrials.gov	157
Total original records	1,310
Bibliographic duplicates removed	333
Other unusable record removed (exact reason unavailable in the retained review record)	1
Original records initially screened	976
Original records excluded at initial screening	938
Original reports assessed at full text	38
Original full-text reports excluded	9

**Table 7:** Reports excluded following the initial full-text assessment and primary reasons for exclusion.

Report	Primary exclusion reason
Blumenthal et al., (2007); PMID: 17846259	Exercise was compared with sertraline/placebo rather than being tested as an adjunct to antidepressant treatment.
Hoffman et al., (2011); PMID: 21148807	Follow-up of exercise versus sertraline/placebo); no eligible adjunctive randomized comparison.
Sherwood et al., (2016); PMID: 26867076	Secondary cardiovascular analysis of exercise versus sertraline/placebo); incorrect randomized comparison and outcome focus.
Heinzel et al., (2022); PMID: 36113693	Exercise was paired with or preceded psychotherapy rather than being tested alongside a common antidepressant regimen.
Szuhany et al., (2020); PMID: 31357916	Psychotherapy augmentation rather than an antidepressant-adjunctive lifestyle intervention design.
García-Toro et al., (2012); PMID: 22516309	Participants with current MDD could not be separated from those with other affective diagnoses.
Salehi et al., (2016); PMID: 26859236	A major co-intervention imbalance prevented attribution of the lifestyle adjunct effect.
Elgendy et al., (2024); PMID: 38540522	No eligible concurrent randomized comparison of depression outcomes.
Schmitter et al., (2025); PMID: 40814823	The antidepressant-treated subgroup was not separately extractable, and pharmacotherapy was not consistent across randomized groups.

**Table 8:** Additional eligible reports identified through supplementary citation searching.

Report	Final eligibility decision
Tavares VD et al. (2025) [37]	Eligible companion report; linked to the existing Tavares/Barros randomized cohort and not double-counted as a new independent study.
Martiny K et al. (2005) [40]	Eligible; report from the same randomized trial as the companion patient-reported publication.
Martiny K et al. (2005) [41]	Eligible companion publication.
Prasko J et al. (2002) [42]	Eligible randomized trial of bright-light therapy and imipramine.
Loving RT et al. (2002) [43]	Eligible randomized trial of bright-light augmentation.
Güzel Özdemir P et al. (2015) [44]	Eligible randomized trial of venlafaxine plus bright-light therapy.
Lam RW et al. (2016) [45]	Eligible four-arm randomized trial; the comparison of bright-light plus fluoxetine versus sham-light plus fluoxetine was relevant.
Chen IP et al. (2022) [46]	Eligible randomized trial comparing adjunctive bright-light therapy with placebo-light therapy.

**Table 9:** Reports excluded during final eligibility verification. MDD: Major depressive disorder.

Report	Reason for exclusion
Martiny K et al. (2012)	Mixed unipolar/bipolar randomized cohort; MDD-specific randomized outcomes were not separately extractable.
Martiny K et al. (2013)	Companion publication from the same ineligible mixed-diagnosis randomized cohort.
Martiny K et al. (2015)	Follow-up companion publication from the same ineligible mixed-diagnosis randomized cohort.

Final study-selection reconciliation: The original searches yielded 38 reports for full-text assessment. Nine were excluded during the original full-text assessment, initially leaving 29 potentially eligible reports. During final eligibility verification, three companion reports from a mixed unipolar/bipolar chronotherapy cohort were excluded because MDD-specific randomized outcomes could not be separately extracted, leaving 26 eligible reports from the original search.

Supplementary citation searching identified eight additional eligible reports. The final review therefore included 34 reports representing 29 independent randomized studies.

All 38 reports counted as full-text assessments were successfully obtained for eligibility assessment. Limitations in access to older literature referred to the incomplete availability of ancillary methodological documentation rather than an inability to retrieve these full-text reports.

**Table 10:** Final study-selection reconciliation n: Number.

Item	n
Original records identified	1,310
Original records removed before screening	334
Original records screened	976
Original records excluded during initial screening	938
Original full-text reports assessed	38
Original full-text reports excluded	9
Additional reports excluded during final eligibility verification	3
Eligible reports remaining from the original search	26
Additional eligible reports identified through supplementary citation searching	8
Final included reports	34
Final independent randomized studies	29

Appendix 3

Quantitative Extraction, Risk of Bias, and GRADE

Detailed quantitative extraction: The extraction table records the randomized sample, intervention and comparator, treatment duration, selected depression outcome, principal numerical result, relevant follow-up, and reported adverse-event or attrition information. Values that were not reported by the original investigators were not reconstructed.

**Table 11:** Detailed quantitative extraction from the included randomized studies AE: Adverse events; BBAT: Basic body awareness therapy; BDI: Beck Depression Inventory; CBT: Cognitive behavioral therapy; ECT: Electroconvulsive therapy; HAM-D/HDRS: Hamilton Depression Rating Scale; IDS-C: Inventory of Depressive Symptomatology-Clinician Rated; MADRS: Montgomery-Åsberg Depression Rating Scale; MBCT: Mindfulness-based cognitive therapy; NNT: Number needed to treat; SSRI: Selective serotonin reuptake inhibitor.

Study	N	Intervention/comparator	Duration	Primary outcome	Quantitative result	Follow-up	Adverse events/attrition
Kuhs H et al. (1996) [13]	51	Amitriptyline plus repeated late partial sleep deprivation versus amitriptyline alone	4 weeks	HAM-D, week 4	Observer-rated improvement and response favored adjunctive sleep deprivation; self-ratings did not confirm superiority.	Not reported	Adverse-event reporting was incomplete.
Reynolds CF 3rd et al. (2005) / Smith GS et al. (2008) [14, 15]	80; PET subset: 16	Total sleep deprivation in a paroxetine/placebo factorial context versus paroxetine/placebo and sleep-procedure comparison conditions	2 weeks	HAM-D, week 2	No sustained acceleration or symptomatic superiority was observed.	2 weeks	Adverse-event reporting was incomplete.
Güdücü F et al. (2005) [16]	37	Sertraline plus partial sleep deprivation or bright-light therapy versus sertraline alone	15 days	HAM-D, day 15	The sleep-deprivation arm showed earlier improvement; the adjunctive arms also showed improvements in anxiety.	15 days	Adverse-event reporting was limited.
Pilu A et al. (2007) / Carta MG et al. (2008) [17, 18]	30	Group physical activity plus ongoing antidepressant treatment versus ongoing antidepressant treatment alone	32 weeks	Clinical depression outcomes, month 8	Adjunctive physical activity favored clinical and functional outcomes; a precise between-group CI was not available for extraction.	32 weeks	Attrition and reporting data were incomplete.
Lavretsky H et al. (2011) [19]	73 randomized partial responders	Tai Chi Chih plus escitalopram versus health education plus escitalopram	10 weeks	HAM-D/remission, week 10	Greater symptom reduction and remission were observed with Tai Chi Chih plus escitalopram.	10 weeks	No major safety signal was highlighted.
Mota-Pereira J et al. (2011) [20]	33	Moderate exercise plus unchanged pharmacotherapy versus pharmacotherapy alone	12 weeks	HAM-D/BDI, week 12	Response and remission rates were 21% and 26%, respectively, versus 0% and 0%; continuous outcomes favored exercise.	12 weeks	Adverse-event reporting was limited.
Schuch FB et al. (2011) [21]	Small inpatient pilot study	Aerobic exercise plus inpatient treatment versus inpatient treatment alone	Hospital stay	HAM-D at discharge	Week 2: 8.2 versus 11.18 (p = 0.192); discharge: 5.93 versus 9.45 (p = 0.041). ECT imbalance limited attribution.	At discharge	No robust adverse-event comparison was available.
Trivedi MH et al. (2011) [22]	126	Higher-dose versus lower-dose exercise augmentation	12 weeks	IDS-C remission, week 12	Adjusted remission rates were 28.3% versus 15.5%; NNT = 7.8; between-dose comparison, p = 0.07.	12 weeks	Adherence and dropout were relevant considerations.
Tanyi RA et al. (2011) [23]	41	Relaxation, guided imagery, and humor plus CBT/pharmacotherapy versus CBT/pharmacotherapy alone	3 weeks	Depression scale, week 3	Within-group improvement was observed; between-group precision and component attribution were limited.	3 weeks	Adverse-event reporting was incomplete.
Danielsson L et al. (2014) [24]	62	Aerobic exercise plus antidepressants, with BBAT as an active intervention arm, versus a single physical-activity consultation/usual care	10 weeks	MADRS, week 10	Reported mean change: −10.3 points (95% CI: −13.5 to −7.1); missing data and expectations limited inference.	10 weeks	No major safety signal was highlighted.
Kerling A et al. (2015) [25]	42	Supervised exercise plus inpatient treatment versus treatment as usual	6 weeks	MADRS/response, week 6	Response was more frequent with exercise; exact event counts were not available for extraction.	6 weeks	Adverse-event reporting was incomplete.
Belvederi Murri M et al. (2015) [26]	121	Progressive or low-intensity exercise plus sertraline versus sertraline alone	24 weeks	HAM-D remission, week 24	Remission rates were 81% with progressive exercise, 73% with low-intensity exercise, and 45% with sertraline alone (p = 0.001).	24 weeks	Complete comparative adverse-event data were not available for extraction.
Carneiro LS et al. (2015) [27]	26 women	Supervised aerobic exercise plus pharmacotherapy versus pharmacotherapy alone	16 weeks	BDI-II/DASS-21, week 16	Depressive symptoms and fitness outcomes favored exercise; the small sample limited precision.	16 weeks	Small sample size.
Shachar-Malach T et al. (2015) [28]	12	Aerobic exercise plus antidepressants versus stretching plus antidepressants	3 weeks	HAM-D response/remission, week 3	Response: 4/6 versus 0/6; remission: 2/6 versus 0/6.	3 weeks	Very small sample size.
Legrand FD and Neff EM (2016) [29]	35	Aerobic exercise plus early antidepressant treatment versus stretching or no intervention plus medication	10 days	BDI-II, day 10	Aerobic exercise versus stretching: Cohen's d = −1.06; no significant improvement was observed in the no-intervention control group.	10 days	Short follow-up duration.
Siqueira CC et al. (2016) [30]	57	Aerobic exercise plus sertraline versus sertraline alone	4 weeks	HAM-D, week 4	Both groups improved; the exercise group received a lower sertraline dose and demonstrated improved fitness.	4 weeks	Detailed adverse-event counts were incomplete.
Moraes HS et al. (2020) [31]	27	Aerobic or strength training plus antidepressants versus low-intensity exercise plus antidepressants	12 weeks	HAM-D/BDI, week 12	Aerobic exercise versus control: HAM-D response, p = 0.044; strength training versus control: BDI response, p = 0.044.	12 weeks	Small sample size.
Haussleiter IS et al. (2020) [32]	111	Guided exercise therapy plus usual inpatient care versus self-organized activity plus usual care	6 weeks	HAM-D, week 6	Guided exercise reduced HAM-D scores more than self-organized activity (p = 0.017).	6 weeks	Detailed comparative adverse-event counts were not available for extraction.
Garcia A et al. (2023) [33]	94	Remote multimodal lifestyle program plus usual treatment versus MBCT or written lifestyle suggestions plus usual treatment	12 months	BDI-II, month 12	No significant between-group benefit was observed for BDI-II scores; secondary lifestyle outcomes improved.	12 months	Attrition was a relevant consideration during the long-term follow-up.
Moreira-Neto A et al. (2024) [34]	40	Aerobic exercise with or without motor complexity plus pharmacotherapy versus no exercise plus pharmacotherapy	24 weeks	Depression/affective outcomes, week 24	Hopelessness and affective outcomes favored exercise; the depression-severity contrast was not the principal efficacy result.	24 weeks	Small pilot study.
Fregna L et al. (2024) [35]	100	Bright-light therapy plus a stable SSRI regimen versus a stable SSRI regimen alone	Acute inpatient course	HAM-D, acute endpoint	Depression improved in both groups; subjective sleep quality improved more clearly with bright-light therapy.	Acute course	Comparative adverse-event estimates lacked precision.
Xu YH et al. (2024) [36]	54	36-hour therapeutic sleep deprivation plus medication versus regular sleep plus medication	7 days	HAM-D24, day 7	Day 3 response rates were 34.8% versus 3.2%; no sustained difference was observed at day 7.	7 days	Supervised setting; missing data were a relevant consideration.
Tavares VD et al. (2025) / Barros ML et al. (2025) [37–39]	59 in the primary efficacy comparison	Exercise plus pharmacotherapy versus pharmacotherapy alone	12 weeks plus follow-up	Depression/remission, week 12	Remission rates were initially 42.3% versus 27.2% and subsequently 72.7% versus 48.1%; the benefit was maintained during follow-up.	4 months	Exercise dropout rate was 15.3%; adverse-event reporting was available in the source.
Martiny K et al. (2005) [40, 41]	102	10,000-lux bright-light therapy plus sertraline 50 mg versus dim red light plus sertraline 50 mg	5 weeks	HAM-D6/HAM-D17, week 5	Clinician-rated depression reduction was significantly greater with bright-light therapy plus sertraline.	5 weeks	Double-blind, placebo-light design.
Prasko J et al. (2002) [42]	34	Bright-light therapy and/or imipramine versus dim red light and/or imipramine comparator arms	3 weeks	HAM-D/SIGH-SAD, week 3	All arms improved; between-group differences were not statistically significant.	3 weeks	Small sample size.
Loving RT et al. (2002) [43]	13 completers	Wake treatment followed by bright-light therapy plus antidepressants versus wake treatment followed by dim red light plus antidepressants	1 week	HDRS-17, week 1	The bright-light arm showed a 27% improvement; between-group significance depended on the exclusion of one placebo outlier.	1 week	Very small sample size.
Güzel Özdemir P et al. (2015) [44]	50	Venlafaxine 150 mg plus bright-light therapy versus venlafaxine 150 mg alone	8 weeks	HDRS, week 8	Week 4 HDRS ≤13: 76% versus 44%; week 8 remission: 76% versus 64% (p = 0.36).	8 weeks	No sham-light control.
Lam RW et al. (2016) [45]	122 total	Bright-light therapy plus fluoxetine 20 mg versus sham device plus fluoxetine 20 mg	8 weeks	MADRS, week 8	Mean change: 16.9 versus 8.8 (p = 0.02); response: 75.9% versus 29.0%; remission: 58.6% versus 19.4%.	8 weeks	Generally well tolerated.
Chen IP et al. (2022) [46]	43	Bright-light therapy plus a stable antidepressant regimen versus dim red placebo light plus a stable antidepressant regimen	4 weeks	HAM-D17, week 4	No group-by-time effect was observed; response: 31.8% versus 33.3%; remission: 13.6% versus 23.8%.	4 weeks	Small sample size.

Outcome- and Timepoint-Specific Cochrane RoB 2 Judgments and Rationales

Overall judgments were classified as low risk, some concerns, or high risk. CAJB completed the primary assessment, and SCJB independently verified the domain judgments and supporting rationales.

RoB 2 judgments were tied to the depression result and assessment timepoint used in the synthesis for each randomized study. Where more than one depression measure, or a severity measure together with response/remission, appears in the outcome label, these co-reported measures refer to the same selected assessment timepoint; no generic study-wide RoB 2 judgment was assigned.

**Table 12:** Outcome- and timepoint-specific Cochrane RoB 2 judgments and rationales. BDI: Beck Depression Inventory; BDI-II: Beck Depression Inventory-II; BLT: Bright-light therapy; D1: Bias arising from the randomization process; D2: Bias due to deviations from intended interventions; D3: Bias due to missing outcome data; D4: Bias in measurement of the outcome; D5: Bias in selection of the reported result; DASS-21: Depression Anxiety Stress Scales-21; ECT: Electroconvulsive therapy; HAM-D: Hamilton Depression Rating Scale; HDRS: Hamilton Depression Rating Scale; IDS-C: Inventory of Depressive Symptomatology-Clinician-Rated; MADRS: Montgomery-Åsberg Depression Rating Scale; RoB 2: Cochrane Risk of Bias 2; SSRI: Selective serotonin reuptake inhibitor; TREAD: Treatment with Exercise Augmentation for Depression.

Study	Outcome/timepoint	D1	D2	D3	D4	D5	Overall	Rationale
Kuhs H et al. (1996) [13]	HAM-D, week 4	Some concerns	Some concerns	Low	Some concerns	Some concerns	Some concerns	Randomization, allocation concealment, and prespecified analyses were incompletely reported; outcome masking was unclear.
Reynolds CF 3rd et al. (2005) / Smith GS et al. (2008) [14, 15]	HAM-D, week 2	Low	Low	Some concerns	Low	Some concerns	Some concerns	The randomized, double-blind design was strong; information regarding attrition, analyses, and selective reporting was not fully reassuring.
Güdücü F et al. (2005) [16]	HAM-D, day 15	Some concerns	Some concerns	Some concerns	Some concerns	Some concerns	Some concerns	Small three-arm trial with limited reporting on allocation concealment, masking, missing data, and prespecified analyses.
Pilu A et al. (2007) / Carta MG et al. (2008) [17, 18]	Clinical depression outcome, month 8	Some concerns	High	High	Some concerns	High	High	Small naturalistic adjunctive trial; deviations from intended interventions, attrition, missing data, and selective outcome reporting materially limited inference.
Lavretsky H et al. (2011) [19]	HAM-D/remission, week 10	Low	Some concerns	Some concerns	Low	Some concerns	Some concerns	Computer-generated randomization and masked outcome assessment reduced bias; behavioral unblinding, attrition, and multiplicity remained concerns.
Mota-Pereira J et al. (2011) [20]	HAM-D/BDI, week 12	Some concerns	Some concerns	Some concerns	Some concerns	Some concerns	Some concerns	Small sample with 2:1 allocation, limited details on allocation and masking, and imprecise categorical outcomes.
Schuch FB et al. (2011) [21]	HAM-D at discharge	Some concerns	High	Some concerns	Low	Some concerns	High	Small inpatient study with unequal ECT cointerventions across randomized groups, directly threatening the treatment contrast.
Trivedi MH et al. (2011) [22]	IDS-C remission, week 12	Low	Some concerns	Some concerns	Low	Low	Some concerns	Randomized dose-comparison trial; adherence and attrition raised some concerns, although the primary remission outcome was prespecified.
Tanyi RA et al. (2011) [23]	Depression scale, week 3	Some concerns	High	Some concerns	High	Some concerns	High	The multicomponent behavioral intervention was unblinded; between-group reporting and outcome measurement were insufficiently robust.
Danielsson L et al. (2014) [24]	MADRS, week 10	Low	Some concerns	Some concerns	Low	Some concerns	Some concerns	Randomized controlled design; missing data, expectation effects, and selective reporting remained potential concerns.
Kerling A et al. (2015) [25]	MADRS/response, week 6	Some concerns	Some concerns	Some concerns	Low	Some concerns	Some concerns	Pilot sample and incomplete reporting of allocation and analyses limited confidence despite clinician-rated outcomes.
Belvederi Murri M et al. (2015) [26]	HAM-D remission, week 24	Low	Some concerns	Some concerns	Low	Low	Some concerns	Randomization and outcome assessment were comparatively strong; adherence and attrition in the exercise arms remained relevant.
Carneiro LS et al. (2015) [27]	BDI-II/DASS-21, week 16	Some concerns	Some concerns	Some concerns	High	Some concerns	High	Very small, unblinded trial with self-reported depression outcomes and limited precision.
Shachar-Malach T et al. (2015) [28]	HAM-D response/remission, week 3	Some concerns	Some concerns	High	Low	High	High	Very small sample (n = 12); missing-data and selective-reporting concerns were substantial despite clinician-rated HAM-D outcomes.
Legrand FD and Neff EM (2016) [29]	BDI-II, day 10	Some concerns	Some concerns	Some concerns	High	Some concerns	High	Small, unblinded trial using the self-reported BDI-II with three behavioral control conditions.
Siqueira CC et al. (2016) [30]	HAM-D, week 4	Low	Some concerns	Low	Low	Low	Some concerns	Randomization and HAM-D assessment were relatively strong; open exercise delivery raised some concerns regarding deviations from intended interventions.
Moraes HS et al. (2020) [31]	HAM-D/BDI, week 12	Some concerns	Some concerns	Some concerns	Low	Some concerns	Some concerns	Small three-arm trial; the low-intensity active control was appropriate, but allocation and missing-data reporting were limited.
Haussleiter IS et al. (2020) [32]	HAM-D, week 6	Some concerns	Some concerns	Some concerns	Low	Some concerns	Some concerns	Active-comparator inpatient trial; allocation, adherence, and prespecified outcome reporting were incompletely described.
Garcia A et al. (2023) [33]	BDI-II, month 12	Low	Some concerns	Some concerns	High	Low	High	Pragmatic behavioral comparison with self-reported BDI-II outcomes and long-term follow-up; masking was not possible, and attrition remained relevant.
Moreira-Neto A et al. (2024) [34]	Depression/affective outcome, week 24	Some concerns	Some concerns	Some concerns	High	Some concerns	High	Pilot trial emphasizing hopelessness and neuroimaging; depression-severity reporting was secondary and incompletely specified.
Fregna L et al. (2024) [35]	HAM-D, acute endpoint	Some concerns	Some concerns	Some concerns	Low	Some concerns	Some concerns	Adjunctive BLT with a stable SSRI regimen; incomplete reporting of allocation and the analysis plan raised some concerns.
Xu YH et al. (2024) [36]	HAM-D24, day 7	Low	Some concerns	High	Low	Low	High	Randomized trial with clinician-rated outcomes; missing outcome data and short follow-up materially affected the day 7 estimate.
Tavares VD et al. (2025) / Barros ML et al. (2025) [37–39]	Depression/remission, week 12	Low	Some concerns	Some concerns	Some concerns	Some concerns	Some concerns	Randomized trial of exercise plus pharmacotherapy; open delivery and attrition remained concerns, but the primary efficacy report was used.
Martiny K et al. (2005) [40, 41]	HAM-D6/HAM-D17, week 5	Low	Low	Some concerns	Low	Some concerns	Some concerns	Randomized, double-blind, placebo-light augmentation trial with identical sertraline treatment; the older trial lacked fully accessible prespecified analysis details.
Prasko J et al. (2002) [42]	HAM-D/SIGH-SAD, week 3	Some concerns	Some concerns	Some concerns	Some concerns	Some concerns	Some concerns	Randomized, double-blind, three-arm study; details regarding allocation concealment, missing-data handling, and the analysis plan were limited.
Loving RT et al. (2002) [43]	HDRS-17, week 1	Some concerns	Some concerns	Some concerns	Some concerns	High	High	Very small trial; between-group significance depended on the exclusion of a placebo outlier, raising substantial selective-analysis concerns.
Güzel Özdemir P et al. (2015) [44]	HDRS, week 8	Some concerns	Some concerns	Low	Some concerns	Some concerns	Some concerns	Randomized comparison involving 25 participants per group; the absence of a sham-light control and unclear assessor masking raised concerns, although attrition appeared limited.
Lam RW et al. (2016) [45]	MADRS, week 8	Low	Low	Some concerns	Low	Low	Some concerns	Registered, double-blind, randomized factorial trial with sham and placebo controls; the modified intention-to-treat requirement for follow-up raised some concerns regarding missing data.
Chen IP et al. (2022) [46]	HAM-D17, week 4	Low	Low	Some concerns	Low	Some concerns	Some concerns	Double-blind, randomized, placebo-light adjunctive trial; small sample size, limited power, and incomplete missing-data information reduced certainty.

GRADE Summary of Findings

Certainty of evidence was assessed separately for clinically coherent outcome domains. Randomized evidence began at high certainty and was downgraded, where appropriate, for risk of bias, inconsistency, indirectness, imprecision, or publication bias.

**Table 13:** GRADE summary of findings. Evidence counts are outcome-specific and may therefore be lower than the total number of studies within each intervention domain, as not every randomized study contributed data to every assessed outcome. Publication bias was considered uncertain because formal assessments of small-study effects were not feasible, and completed but unpublished trials identified through trial registries could not be reliably classified retrospectively. GRADE: Grading of Recommendations Assessment, Development and Evaluation; RCT: Randomized controlled trial.

Outcome/domain	Evidence	Risk of bias	Inconsistency	Indirectness	Imprecision	Publication bias	Certainty	Explanation
Exercise/mind-body: depressive-symptom severity	15 RCT clusters	Serious	Serious	Not serious	Not serious	Uncertain	Low	Most trials favored structured exercise, but findings from active-control and pragmatic comparisons were less consistent.
Exercise: response/remission	10 RCT clusters	Serious	Not serious	Not serious	Serious	Uncertain	Low	Several favorable estimates were reported; however, small event counts and imprecision limited confidence.
Sleep/circadian/bright-light interventions: depression outcomes	11 RCT clusters	Serious	Serious	Not serious	Serious	Uncertain	Very low	Positive and neutral findings were reported across trials; intervention modalities and assessment timepoints differed, and many studies were small or of short duration.
Multicomponent lifestyle: depression severity	2 RCT clusters	Serious	Serious	Not serious	Serious	Uncertain	Very low	Two heterogeneous studies provided inconsistent and imprecise evidence.
Adverse events/acceptability	Across 29 RCT clusters	Serious	Not serious	Serious	Serious	Uncertain	Very low	Reporting of adverse events was incomplete and inconsistent.
Whole-diet-only adjunct	0 eligible RCTs	-	-	-	-	-	Not assessable	No eligible randomized trial was identified.
